# Microbe-Derived Indole Metabolite Demonstrates Potent Multidrug Efflux Pump Inhibition in *Staphylococcus aureus*

**DOI:** 10.3389/fmicb.2019.02153

**Published:** 2019-09-18

**Authors:** Rushikesh Tambat, Manoj Jangra, Nisha Mahey, Nishtha Chandal, Manpreet Kaur, Surbhi Chaudhary, Dipesh Kumar Verma, Krishan Gopal Thakur, Manoj Raje, Sanjay Jachak, Neeraj Khatri, Hemraj Nandanwar

**Affiliations:** ^1^Clinical Microbiology and Bioactive Screening Laboratory, CSIR – Institute of Microbial Technology, Chandigarh, India; ^2^Cell Biology and Microscopy Laboratory, CSIR – Institute of Microbial Technology, Chandigarh, India; ^3^Structural Biology Laboratory, CSIR – Institute of Microbial Technology, Chandigarh, India; ^4^Department of Natural Products, National Institute of Pharmaceutical Education and Research, Mohali, India; ^5^Animal House Facility, CSIR – Institute of Microbial Technology, Chandigarh, India

**Keywords:** multidrug resistance, efflux pump inhibitor, time-kill kinetics, post-antibiotic effect, membrane potential, combination therapy, natural products

## Abstract

Efflux pumps are always at the forefront of bacterial multidrug resistance and account for the failure of antibiotics. The present study explored the potential of 2-(2-Aminophenyl) indole (RP2), an efflux pump inhibitor (EPI) isolated from the soil bacterium, to overcome the efflux-mediated resistance in *Staphylococcus aureus*. The RP2/antibiotic combination was tested against efflux pump over-expressed *S. aureus* strains. The compound was further examined for the ethidium bromide (EtBr) uptake and efflux inhibition assay (a hallmark of EPI functionality) and cytoplasmic membrane depolarization. The safety profile of RP2 was investigated using *in vitro* cytotoxicity assay and Ca^2+^ channel inhibitory effect. The *in vivo* efficacy of RP2 was studied in an animal model in combination with ciprofloxacin. RP2 exhibited the synergistic activity with several antibiotics in efflux pump over-expressed strains of *S. aureus*. In the mechanistic experiments, RP2 increased the accumulation of EtBr, and demonstrated the inhibition of its efflux. The antibiotic-EPI combinations resulted in extended post antibiotic effects as well as a decrease in mutation prevention concentration of antibiotics. Additionally, the *in silico* docking studies suggested the binding of RP2 to the active site of modeled structure of NorA efflux pump. The compound displayed low mammalian cytotoxicity and had no Ca^2+^ channel inhibitory effect. In *ex vivo* experiments, RP2 reduced the intracellular invasion of *S. aureus* in macrophages. Furthermore, the RP2/ciprofloxacin combination demonstrated remarkable efficacy in a murine thigh infection model. In conclusion, RP2 represents a promising candidate as bacterial EPI, which can be used in the form of a novel therapeutic regimen along with existing and upcoming antibiotics, for the eradication of *S. aureus* infections.

## Introduction

Methicillin-resistant *Staphylococcus aureus* (MRSA), a high priority pathogen (Tacconelli et al., 2018), is responsible for skin and soft tissue infections, endocarditis, and several bloodstream infections (Tong et al., 2015). For the last few decades, there is a substantial decline in the development of new antimicrobial compounds (Boucher et al., 2009). With diminished hope on dwindling discovery pipeline, bridging the gap between widespread multi-drug resistance, and development of new antibiotics requires innovative use of available antibiotic arsenal. Resistance-modifying agents (Abreu et al., 2012) can be used as antibiotic potentiators in combination therapy for the development of novel treatment regimens.

The over-expression of efflux pumps in *S. aureus* is one of the major cause of antibiotic resistance (Munita and Arias, 2016). Several efflux pumps have been reported in bacteria, of which major facilitator superfamily (MFS) is predominant (Costa et al., 2013), which utilizes a proton gradient for extruding the drugs. In *S. aureus*, NorA (MFS) is the most studied efflux pump and is reported to be over-expressed in more than 50% of the clinical isolates (DeMarco et al., 2007). The NorA pump exports a broad range of compounds such as fluoroquinolones, detergents, and several dyes like ethidium bromide (EtBr) (Fontaine et al., 2015; Tintino et al., 2016), whereas TetK and MsrA efflux pumps are specific for tetracycline and erythromycin, respectively (Smith et al., 2007).

No efflux pump inhibitor/antibiotic cocktail has been approved hitherto, possibly due to the unnecessary toxicities in human (Stavri et al., 2006). To the best of our knowledge, however, no systematic study has been carried out in the past for EPIs isolated from microbes against *S. aureus* strains. To compete with the resistance mechanism, antibiotic-producing bacteria have the tendency to counterattack by means of some inhibitors (Wright, 2014). Therefore, the microbes represent a unique bioprospecting opportunity, because there is substantial evidence that bacteria produce inhibitors against efflux pumps (Lee et al., 2001; Whalen et al., 2015).

The current study describes the isolation, identification and assessment of efflux pump inhibitory activity of EPI 2-(2- Aminophenyl) indole from the terrestrial bacterial isolate, *Streptomyces* Sp. IMTB 2501. The compound was further evaluated for the *in vitro* toxicity. Additionally, we checked the impact of RP2 on invasive abilities of *S. aureus* inside macrophages and in a neutropenic murine thigh infection model. The results obtained in this study may aid the development of RP2 as potent EPI against *S. aureus* efflux pumps.

## Materials and Methods

### Chemicals, Bacterial Strains, and Growth Conditions

All the antibiotics, chemicals, reagents and dyes used in this study were purchased from Sigma-Aldrich Chemical Co. (St. Louis, MO, United States) unless mentioned otherwise. The strains, SA-1199B (NorA over-expressed) (Kaatz et al., 1987), SA-K1758 (NorA deletion mutant of NCTC 8325-4) (Price et al., 2002), XU212 (clinical isolate, TetK over-expressed) (Gibbons and Udo, 2000), and RN4220-MsrA (transformed with pSK265, containing MsrA efflux gene) (Reynolds et al., 2003) were used in the study. *S. aureus* ATCC 25923 (Himedia^®^) was used in mutation studies. Cation-adjusted Mueller-Hinton broth (CA-MHB; Becton-Dickinson, United States) was used to grow the strains.

### Purification and Characterization of Bioactive Metabolite

The microbial extract library preparation and screening platform for EPI activity is discussed in Supplementary Material. The 48 h old inoculum of soil isolate IMTB 2501 was grown in R-2A broth (Himedia^®^, India), supplemented with 1% NaCl, and 0.2% calcium carbonate. After 96 h of incubation, cells were removed by centrifugation and extract was prepared using Diaion^®^ HP-20 resin. The crude extract was fractionated on Sephadex^TM^ LH-20 (GE Healthcare) column using a step gradient of methanol: water. The active fractions were pooled and processed further by reverse-phase high-pressure liquid chromatography (RP-HPLC) (Waters, XBridge^®^ BEH C18 OBD^TM^ Prep Column 130 Å, 5 μm, 10 mm × 250 mm). The mobile phase consisted of solvent A as 10 mM ammonium acetate (pH 5.5) and solvent B as 100% acetonitrile. The flow rate was kept constant at 3 mL/min. The following gradient was used; 5% solvent B for 5 min, 5 to 95% B in 45 min, hold at 95% B for 10 min. The peaks were collected and assayed for the bioactivity. The active peak (termed as RP2) was identified and subjected to lyophilization. The purified compound was subjected to gas chromatography (Varian 450-GC) coupled with mass spectrometry (Varian 20-MS). The spectrum was recorded in an electron-ionization mode. NMR spectra (^1^H, ^13^C, DEPT, COSY, and HSQC) were recorded on a Bruker 400 MHz spectrometer in DMSO-*d*_6_.

### Checkerboard Synergy Assay

To examine the synergistic activity of RP2 with the antibiotics, the checkerboard assay was performed. The MIC values of the combinations were used to calculate the fractional inhibitory concentration (FICI) index according to the formula:

FICI=MIC(antibioticcombinedwithcompound)/MIC⁢(antibiotic⁢alone)+MIC(compoundcombinedwithantibiotic)/MIC⁢(compound⁢alone)

The FICI value of ≤0.5 was considered as “Synergy” whereas 0.5 < FICI ≤ 4.0 was observed as an “indifference” effect and the FICI > 4.0 was considered as “antagonistic” effect (Junio et al., 2011).

### Ethidium Bromide Accumulation Assay

The fluorometric estimation of EtBr uptake was performed in *S. aureus* SA-1199B, XU212, and RN4220-MsrA as described previously (Kalia et al., 2012). *S. aureus* cells (OD_550_ ∼0.2) were treated with EtBr at 2 μg/mL in the presence of RP2 at its 1/4th MIC (32 μg/mL) or reserpine (20 μg/mL). The accumulation of fluorescence was recorded over a period of 45 min at 3-min intervals at excitation and emission wavelength of 530 and 600 nm, respectively, in a microplate reader (BioTek, United States).

### Ethidium Bromide Efflux Inhibition Studies

The effect of the RP2 on EtBr efflux of *S. aureus* SA-1199B, XU212 and RN4220-MsrA was assessed by fluorometry (Viveiros et al., 2008). A 50 μl of the cell suspension (OD_600_ ∼0.6) was incubated with 50 μl of phosphate buffer saline (PBS) containing EtBr (2 μg/mL) and RP2 (4–32 μg/mL) or reserpine (20 μg/mL). Post 30 min incubation, the fluorescence was acquired at 530/600 nm. Relative final fluorescence (RFF) index was calculated according to the formula: RFF = (RF_treated_ – RF_untreated_)/RF_untreated_ where the RF_treated_ denotes the final fluorescence at the last time point in the presence of RP2 or reserpine; the RF_untreated_ denotes the final fluorescence at the last time point (45 min) of the experiment in the control well (without EPI) (Machado et al., 2011).

### Time-Kill Kinetics

*Staphylococcus aureus* SA-1199B (10^6^ colony forming units; CFU/mL) was incubated with ciprofloxacin in the presence or absence of RP2 (32 μg/mL). At different time points, the culture was spread plated and the colonies were expressed as CFU/mL. The sample without antibiotic and RP2 was taken as a positive control.

### Mutation Prevention Concentration (MPC)

*Staphylococcus aureus* ATCC 25923 (10^8^ CFU) was plated on MHA plates containing different concentrations of ciprofloxacin, tetracycline or erythromycin. The similar concentrations of antibiotics were also tested in the presence of RP2 (16 and 32 μg/mL). After 48 h, the MPC was determined as the concentration at which no colony appeared. The mutation frequency was calculated as the number of survivors divided by CFU plated (Drugeon et al., 1999).

### Post-antibiotic Effect (PAE)

*Staphylococcus aureus* SA-1199B, XU212, and RN42220 (2 × 10^8^ CFU/mL) were treated with ciprofloxacin, tetracycline, and erythromycin, respectively, at different concentrations, with or without RP2. Post 2 h of incubation, the culture was diluted 1000 times in fresh medium to remove the effect of antibiotics and RP2, followed by determination of viable CFU/mL at different time points. The difference in the time taken for growth between treated culture (T) and the equivalent untreated control (C) to escalate by 1 log_10_ CFU/mL, was calculated as PAE = T – C (Craig, 1991).

### Structure Prediction of NorA and Docking Studies With RP2

The three dimensional structure of NorA protein was predicted using I-TASSER online server (Roy et al., 2010) using PDB IDs 3WDO, 6EXS, 4ZOW, and 4ZPO as templates. This modeled NorA receptor was further used for the docking studies. The ligand and receptor preparation and molecular docking studies were carried out using Schrodinger Suite 2019. The two-dimensional structure of the RP2 was prepared using Maestro. The receptor was prepared for docking using the protein preparation wizard (Sastry et al., 2013) by adding hydrogen atoms to the receptor molecule followed by energy minimization using OLPS3 force field by keeping default 0.3 Å root mean square deviation (RMSD) constraint (Shivakumar et al., 2012). The potential RP2 binding sites in the modeled structure were identified using the SiteMap (Halgren, 2007). The SiteMap analysis identified one potential druggable site with site score of >1 located at the active site of predicted NorA structure. This site was used for grid generation. The grid was generated for docking by keeping box dimension 40 Å encompassing all the protein structure. The molecular docking was performed using Induced Fit Docking module (Farid et al., 2006). The poses were sorted based on the docking score.

### Membrane Potential Assay

The membrane depolarization effect of RP2 was quantified using the *BacLight*^TM^ Bacterial Membrane Potential Kit (Molecular Probes, Life Technologies). *S. aureus* SA-1199B (10^7^ CFU/mL) was treated with RP2 at concentrations ranging from 4 to 32 μg/mL or carbonyl cyanide 3-chlorophenylhydrazone (CCCP, 2 μg/mL). The dye, 3, 3′-Diethyloxacarbocyanide (DiOC_2_) was added and the plates were incubated for 30 min. The fluorescence was recorded and change in membrane potential was evaluated.

### Determination of Intracellular ATP Levels

The levels of intracellular ATP were evaluated using the ATP Determination Kit (Invitrogen, Life Technologies, United Kingdom) (Machado et al., 2017). *S. aureus* SA-1199B (10^5^ CFU/mL) was incubated with RP2 (32 μg/mL) in MHB. At different time intervals, the cells were lysed by alternative heat and cold shock method and the supernatant was used for the measurement of total ATP, represented as relative luminescence units.

### Mammalian Ca^2+^ Channel Blocking Assay

Fluo-4 Direct calcium channel assay kit (Life Technologies, Carlsbad, CA, United States) was used, to check the effect of RP2 on human Ca^2+^ channels (Blanchard et al., 2014). Fluo-4 dye containing probenecid (5 mM) was incubated with HEK 293T cells (5 × 10^4^) for 1 h and fluorescence was measured for 15 s. At 15 s, the cells were treated separately with DMSO (mock), 50 μg/mL of verapamil, or 32 μg/mL of RP2. Then, the Ca^2+^ channel stimulator carbamylcholine chloride (50 μg/mL) was added at 60 s and the fluorescence was quantified for another 120 s on a microplate reader.

### Mammalian Cytotoxicity and Hemolytic Activity

The cytotoxicity of RP2, at a concentration ranging from 62.5 to 1000 μg/mL, was investigated in three cell lines viz; THP-1 (Human Monocytic cell line), HEK-293 (human embryonic kidney cell line) and Hep G2 (human liver cancer cell line). The hemolysis assay was performed with fresh rabbit erythrocytes, as described previously (Jangra et al., 2019).

### Macrophage Invasion Assay

The effect of RP2 on the intracellular invasion of *S. aureus* inside the J774 macrophage cell line was determined (Kalia et al., 2012). *S. aureus* strains, SA-1199B, SA-1199, and K1758 (10^6^ CFU/well) were used to infect the macrophage (10^5^ cells/well) in the presence or absence of RP2 (32 μg/mL) and incubated for 2 h. The extracellular bacteria were removed with PBS and the cells were further treated with gentamicin (50 μg/mL) for 30 min. Intracellular bacteria were rescued by host cell denaturation using a short exposure of 0.1% saponin and the viability was determined by plating on MHA.

### Neutropenic Thigh Infection Model

Female BALB/c mice were made neutropenic by an intraperitoneal administration with cyclophosphamide at 4 (150 mg/kg) and 1 (100 mg/kg) days before infection. An inoculum of 10^7^ CFU/mL *S. aureus* SA-1199B was resuspended in sterile PBS followed by 50 μL intramuscular injection into the right thighs of mice. At 4 h after infection, one group was euthanized by cervical dislocation. The right thighs were aseptically removed, homogenized in PBS, serially diluted and plated on drug-free MHA for CFU counts. The remaining mice were treated with either RP2 or ciprofloxacin (both at 10 mg/kg) alone or in combination, administered subcutaneously (six mice per group). At 24 h after infection, the mice were sacrificed and CFUs were calculated in the thigh muscle (Ling et al., 2015).

### Statistical Analysis

Statistical analysis was carried out using the Student’s *t*-test. A *P* value < 0.05 was considered statistically significant and highly significant when ^∗∗^*P* < 0.01 and ^∗∗∗^*P* < 0.001.

## Results

### Isolation and Identification of 2-(2-Aminophenyl) Indole

**FIGURE 1:**
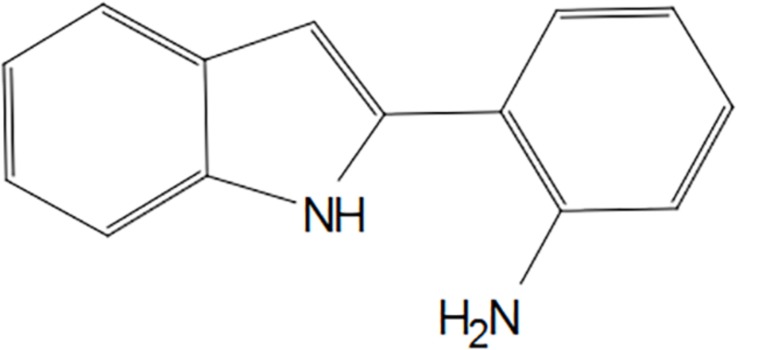
2D structure of RP2.

The initial screening identified 50 microbial isolates (Supplementary Table S1), which potentiated the activity of EtBr in *S. aureus* by at least four folds. Among them, eight isolates (IMTB 2342, IMTB 2501, IMTF 935, IMTF 1118, IMTF 1984, IMTF 2261, IMTF 2413, and IMTF 2454) reduced the MIC of at least one of the antibiotic tested, i.e., norfloxacin, erythromycin and tetracycline by ≥fourfold against *S. aureus* SA-1199B, XU212 and RN4220-MsrA, respectively (Supplementary Table S2). The isolate IMTB 2501 exhibited the most prominent EPI-like activity and was selected for further study. This strain showed 98.90% similarity with *Streptomyces roseochromogenes* based on 16S rRNA gene sequence (GenBank accession number MK377074). Bioactivity-guided fractionation of the microbial extract of IMTB 2501 resulted in the isolation of 2-(2- Aminophenyl) indole (C_14_H_12_N_2_) (Figure 1), designated as “RP2.” The structure of RP2 was elucidated on the basis of GC-MS and NMR spectroscopic data (Supplementary Figures S1–S4 and Supplementary Table S3). This article represents the first description of 2-(2-Aminophenyl) indole from a microbial source, with novel EPI functionality.

### Checkerboard Synergy Assay

RP2 exhibited no intrinsic antibacterial activity up to 32 μg/mL, against *S. aureus* SA-1199B, SA-K1758, XU212 and RN4220-MsrA strains. At this concentration, it reduced the MICs of norfloxacin, ciprofloxacin, moxifloxacin, and chloramphenicol in SA-1199B by 64, 16, 4, and 4 folds, respectively (Table 1), indicating the synergy (FICI ≤ 0.5). On the contrary, SA-K1758 did not show any significant reduction in the MIC. The low MIC value of moxifloxacin in SA-1199B indicates that it is presumably a weaker substrate of NorA efflux pump. RP2 also inhibited TetK and MsrA transporters and reduced the MICs of tetracycline in *S. aureus* XU212 and erythromycin in RN4220-MsrA by 64 (FICI 0.265) and 32 (FICI 0.281) folds, respectively (Table 1). We also checked synergy of norfloxacin, tetracycline and erythromycin with reserpine, a known efflux pump inhibitor. Reserpine modulated the MICs of norfloxacin and tetracycline by eight- and four-fold, respectively, whereas no synergy was observed with erythromycin (Supplementary Table S4). These results depicted that RP2 also displayed broad efflux pump inhibition spectrum.

**TABLE 1:** MICs for numerous antibiotics against *S. aureus* efflux pump over-expressed/deletion strains in the absence and presence of RP2.

**Compounds**	***S. aureus* SA1199B (NorA^++^)**			***S. aureus* K1758 (NorA^–^)**		
	**MIC^a^**	**MIC^b^**	**FICI^c^**	**MIC^a^**	**MIC^b^**	**FICI^c^**
Norfloxacin	32	0.5 (32)	0.265	0.25	0.125 (16)	0.75
		2 (16)	0.1875		0.25 (8)	–
		4 (8)	0.1875		0.25 (4)	–
		8 (4)	0.281		0.25 (2)	–
Ciprofloxacin	8	0.5 (32)	0.3125	0.125	0.125 (16)	–
		1 (16)	0.25		0.125 (8)	–
		4 (8)	0.5625		0.125 (4)	–
Moxifloxacin	0.25	0.0625 (32)	0.375	0.0625	0.0625 (16)	–
		0.125 (16)	0.625		0.0625 (8)	–
Chloramphenicol	8	2 (32)	0.375	2	2 (16)	–
		4 (16)	0.625		2 (8)	–
RP2	128	n/a	n/a	64	n/a	n/a

	***S. aureus* XU212 (TetK)^∗^**			***S. aureus* RN4220-MsrA^∗∗^**		

Tetracycline^1^/Erythromycin^2^	128	2 (32)	0.265	128	4 (32)	0.281
		4 (16)	0.156		16 (16)	0.25
		8 (8)	0.125		32 (8)	0.3125
		32 (4)	0.281		64 (4)	0.531
RP2	128	n/a	n/a	128	n/a	n/a

*MIC^*a*^, minimum inhibitory concentration of compound alone (μg/mL); MIC^*b*^, MIC of antibiotic in the presence of RP2 (μg/mL in parentheses); FICI^*c*^, fractional inhibitory concentration index. The checkerboard synergy assay of RP2 in combination with Tetracycline^1^ and Erythromycin^2^ was performed against *S. aureus* XU212^∗^ and *S. aureus* RN4220-MsrA^∗∗^, respectively.^++^, NorA over-expressed strain; ^––^, NorA deletion strain.*

### Effect of RP2 on Accumulation and Efflux of EtBr

**FIGURE 2:**
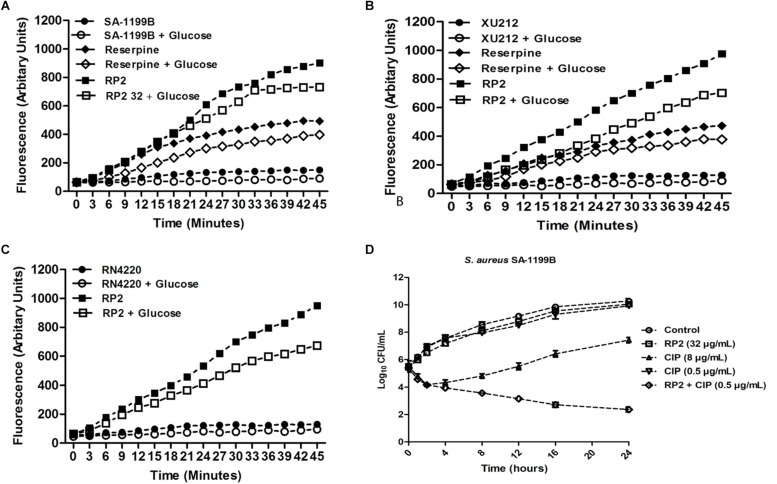
Effect of RP2 on the accumulation of ethidium bromide by *S. aureus*: **(A)** SA-1199B, **(B)** XU212, and **(C)** RN4220-MsrA; the concentration of EtBr was 2 μg/mL for all the three tested strains. Assays were performed at 37°C in the presence and absence of glucose. Reserpine was used at 20 μg/mL as the positive control for *S. aureus* SA-1199B and XU212. **(D)** Time-kill curve of *S. aureus* SA-1199B, showing the bactericidal effect of ciprofloxacin in combination with RP2 at 32 μg/mL. Each time point represents the mean log_10_ CFU ± SD of three independent readings.

The expression of NorA (SA-1199B), TetK (XU212), and MsrA (RN4220-MsrA) efflux pumps resulted in a slow increase in fluorescence, indicating the negligible accumulation of EtBr (Figures 2A–C). The addition of RP2 enhanced the accumulation significantly, compared to reserpine, a known NorA, and TetK efflux inhibitor (Smith et al., 2007). The presence of glucose reduced the efflux pump inhibitory activity of both RP2 and reserpine, because glucose reenergizes the cells, promoting active efflux. We next evaluated the potential of RP2 in these strains, to inhibit EtBr efflux, and calculated the RFF values (Table 2). The RFF is a measure of efflux inhibition by comparing the final fluorescence of EPI treated vs. untreated cells. The RFF value >1 indicates an enhanced accumulation of EtBr (Machado et al., 2017). At 32 μg/mL, RP2 displayed the RFF values of 11.51, 9.39 and 8.69 against the NorA, TetK, and MsrA efflux pump over-expressed strains, respectively, in comparison to RFF values of reserpine, 6.22 (NorA) and 2.97 (TetK). RP2 demonstrated dose-dependent EtBr accumulation and efflux inhibition.

**TABLE 2:** Relative Final Fluorescence (RFF) values based on the EtBr efflux inhibition for the *S. aureus* strains in the presence of the efflux inhibitors.

**Compound**	**Conc. of inhibitor used (μg/mL)**	**Relative final fluorescence (RFF ± SD)**		
		**SA1199B**	**XU212**	**RN4220-MsrA**
RP2	32	11.51 ± 0.16^∗∗∗^	9.39 ± 0.08^∗∗∗^	8.69 ± 0.12^∗∗∗^
	16	8.9 ± 0.14^∗∗∗^	6.8 ± 0.03^∗∗∗^	6.04 ± 0.12^∗∗∗^
	8	7.27 ± 0.14^∗∗∗^	4.61 ± 0.11^∗∗∗^	4.58 ± 0.06^∗∗∗^
	4	5.06 ± 0.07^∗∗∗^	2.86 ± 0.08^∗∗^	3.03 ± 0.1^∗∗^
Reserpine	20	6.22 ± 0.11^∗∗∗^	2.97 ± 0.05^∗∗^	ND^a^

*The results presented correspond to the average of three independent assays plus standard deviation (±SD). Results were considered highly significant when ^∗∗^*P* < 0.01 and ^∗∗∗^*P* < 0.001. ^*a*^ND, not determined.*

### Time-Kill Kinetics and Mutation Frequency Analysis

The time-kill kinetics of *S. aureus* SA-1199B was examined with the combination of ciprofloxacin (8 μg/mL) and RP2 (32 μg/mL). As shown in Figure 2D, the combination resulted in a reduction of ≥2 log_10_ CFU in 24 h, in comparison to the antibiotic or RP2 alone. These results prompted us to study the frequency of mutation in *S. aureus* in the presence of RP2-antibiotic combinations. We observed that RP2 at 16 μg/mL decreased the MPC of ciprofloxacin, tetracycline and erythromycin by twofold each, whereas by 8, 8, and 4 folds, respectively when tested at 32 μg/mL (Table 3). These results highlight the clinical significance of these combinations in limiting the selection of resistant mutants.

**TABLE 3:** Mutation frequencies of *S. aureus* ATCC 25923.

**RP2 (μg/mL)**	**Mutation frequency**											
	**Ciprofloxacin**				**Tetracycline**				**Erythromycin**			
	**2 MIC**	**4 MIC**	**8 MIC**	**16 MIC**	**2 MIC**	**4 MIC**	**8 MIC**	**16 MIC**	**2 MIC**	**4 MIC**	**8 MIC**	**16 MIC**
0	5.5 × 10^–8^	3.9 × 10^–8^	1.8 × 10^–8^	<10^–8^	5.6 × 10^–8^	4.8 × 10^–8^	2.5 × 10^–8^	<10^–8^	6.9 × 10^–8^	5.1 × 10^–8^	3.5 × 10^–8^	<10^–8^
16	3.6 × 10^–8^	1.5 × 10^–8^	<10^–8^	<10^–8^	2.7 × 10^–8^	1.3 × 10^–8^	<10^–8^	<10^–8^	3.6 × 10^–8^	1.4 × 10^–8^	<10^–8^	<10^–8^
32	<10^–8^	<10^–8^	<10^–8^	<10^–8^	<10^–8^	<10^–8^	<10^–8^	<10^–8^	1.2 × 10^–8^	<10^–8^	<10^–8^	<10^–8^

*The results presented correspond to the average of three independent assays.*

### Post-antibiotic Effect

The post-antibiotic effect is the process of continued suppression of bacterial growth after a short exposure to antibiotics (Craig, 1991). In the absence of RP2, the PAEs at a 1 × MIC concentration of ciprofloxacin (*S. aureus* SA-1199B), tetracycline (XU212), and erythromycin (RN4220-MsrA) were 1.43, 2.1, and 1.82 h, respectively (Table 4). The same concentrations of antibiotics in combination with RP2 (32 μg/mL) resulted in significantly prolonged PAEs, ciprofloxacin (3.33 h), tetracycline (3.4 h), and erythromycin (3.32 h). Similar effects were obtained at lower concentrations of antibiotics.

**TABLE 4:** PAE of ciprofloxacin, tetracycline, and erythromycin alone and in combination with RP2 against *S. aureus* SA1199B, XU212 and RN4220-MsrA, respectively.

**Strain**	**Regimen**	**Mean PAE (h) ± SD**		
		**0.25 MIC**	**0.5 MIC**	**1 MIC**
*S. aureus* SA1199B	Ciprofloxacin	0.43 ± 0.14	1.05 ± 0.19	1.43 ± 0.16
	Ciprofloxacin + RP2 (32 μg/mL)	1.68 ± 0.13	2.15 ± 0.15	3.33 ± 0.18
*S. aureus* XU212	Tetracycline	0.76 ± 0.13	1.2 ± 0.17	2.1 ± 0.14
	Tetracycline + RP2 (32 μg/mL)	1.93 ± 0.12	2.45 ± 0.08	3.4 ± 0.25
*S. aureus* RN4220-MsrA	Erythromycin	0.46 ± 0.13	1.08 ± 0.17	1.82 ± 0.14
	Erythromycin + RP2 (32 μg/mL)	1.77 ± 0.12	2.24 ± 0.08	3.32 ± 0.25

*The results presented correspond to the average of three independent assays plus standard deviation (±SD).*

### *In silico* Docking Studies of RP2 With NorA Efflux Pump Model

**FIGURE 3:**
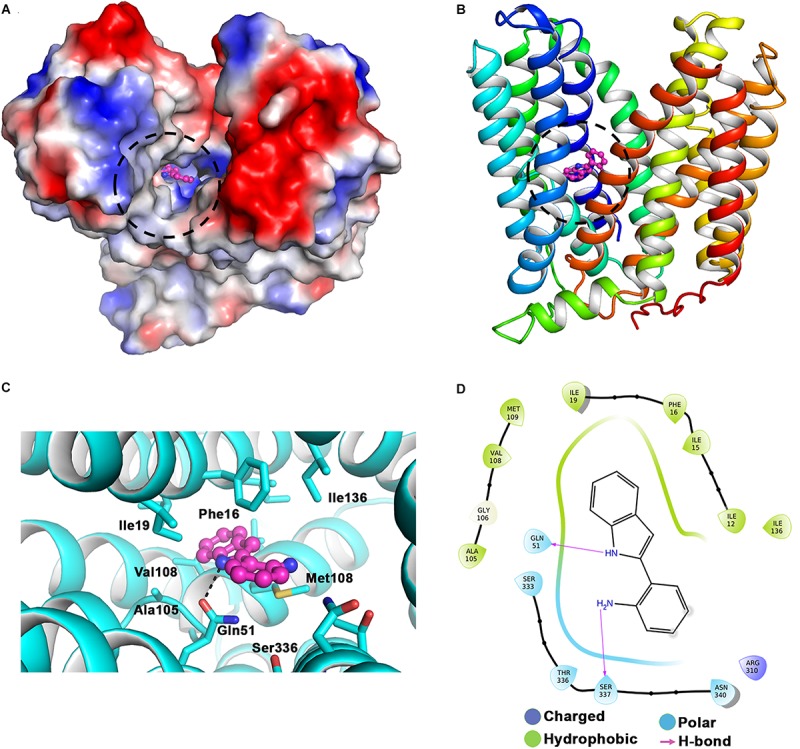
Interaction of RP2 with the active site of NorA. **(A)** 3D electrostatic potential map and **(B)** cartoon representation (in rainbow color) of NorA showing RP2 (ball and stick representation in magenta) docked in the active site cleft. **(C)** The close view of NorA residues, in stick representation, and interacting with RP2. All 12 transmembrane helices of the protein have been colored in rainbow. **(D)** Ligand interaction diagram in 2D representation showing several interactions involved in NorA/RP2 binding.

The predicted structure of NorA consists of 12 transmembrane helices with a wide cavity at the active site located in the center (Figures 3A,B). We used box size of 40 Å encompassing all the protein structure for docking RP2 to identify the potential binding site. 57 out of 65 poses of the docked structure were observed at site 1 and the remaining 8 poses were observed at site 2 (Figure 3 and Supplementary Figures S5A,B). The docking scores for the poses observed in the site 1 were better (<−7.8) compared to the site 2 (>−6.7), therefore we only selected site 1 for further interaction analysis. The docking results suggested that RP2 binding is stabilized by hydrophobic interactions, H-bonding and/or Pi–Pi stacking interactions. The RP2/NorA binding site is lined by two conserved hydrophobic patches at the one end and a hydrophilic patch which covers the other end (Figure 3). These two hydrophobic patches are lined by Ile12, Ile15, Phe16, Leu19, and Ala105, Gly106, Val108, Met109 residues. The hydrophilic patch is composed of Ser333, Thr336, Ser337 and Asn340 residues. Analysis of all 65 different poses suggested that in several poses Gln51 participated in hydrogen bonding with the amino group of RP2. Interestingly, Gln51 has been shown to be involved in stabilizing NorA/inhibitor binding in previous studies as well. In some poses, we also observed Pi-Pi stacking interactions between Phe16/Phe47or Phe140 with benzene ring of RP2. The variations observed in the mode of binding and natures of interactions stabilizing RP2 in selected docked poses are shown in the Supplementary Figures S5C–G. The comparative analysis of RP2 binding with other known NorA inhibitors (analyzed by virtual docking) like ferruginol, totarol, thioxanthene, phenothiazine, levofloxacin, and acridine suggested that they also bind at the same site and share several common interacting residues (Bhaskar et al., 2016). However, unlike other inhibitors, reserpine interacts with only one of the conserved hydrophobic clefts (Ala105, Gly106, Val108, and Met109) (Kalia et al., 2012).

### Membrane Depolarization

**FIGURE 4:**
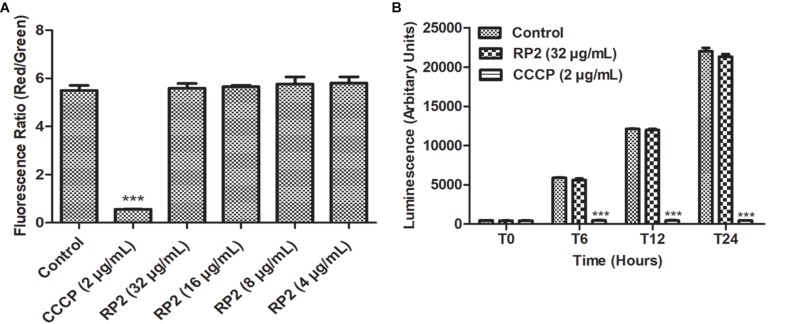
**(A)** Membrane depolarization. The relative red/green ratio of *S. aureus* SA-1199B using DIOC_2_ stained cells after 30 min of exposure to RP2 from 4 to 32 μg/mL. Green and red fluorescence correspond to the depolarized as well as polarized cells, respectively. The changes in the fluorescence were measured at an excitation/emission wavelength of 485/528 nm and 528/590 for green and red fluorescence, respectively. **(B)** Effect of RP2 on *S. aureus* ATP levels. *S. aureus* SA-1199B was exposed to RP2 at 1/4th MIC during 24 h. The ATP levels were quantified using a luciferin-luciferase bioluminescence detection assay. CCCP (2 μg/mL) was included for comparison. The results presented correspond to the mean of three independent readings ± SD. Results were considered highly significant where ^∗∗∗^*P* < 0.001.

Thirty minutes after the addition of RP2 (4–32 μg/mL), there was no significant change in the percentage of depolarized cells when compared with untreated bacterial cells, indicating no interference of bacterial membrane potential by RP2 (Figure 4A). In contrast, the protonophore CCCP distorted the membrane potential. The percentage of depolarized cells was >90%.

### Effect of RP2 on Intracellular ATP Levels

The effect of RP2 on bacterial ATP synthesis was assessed in *S. aureus* SA-1199B at a sub-inhibitory concentration (32 μg/mL). The ATP levels remained unaffected during 24 h of exposure with RP2, similar to that of drug-free control (Figure 4B). These results excluded the possibility of EtBr accumulation and efflux inhibition due to sudden ATP depletion. In contrast, the impairment in ATP production was observed when cells were treated with CCCP (positive control) at 2 μg/mL.

### Mammalian Ca^2+^ Channel Blocking Assays

**FIGURE 5:**
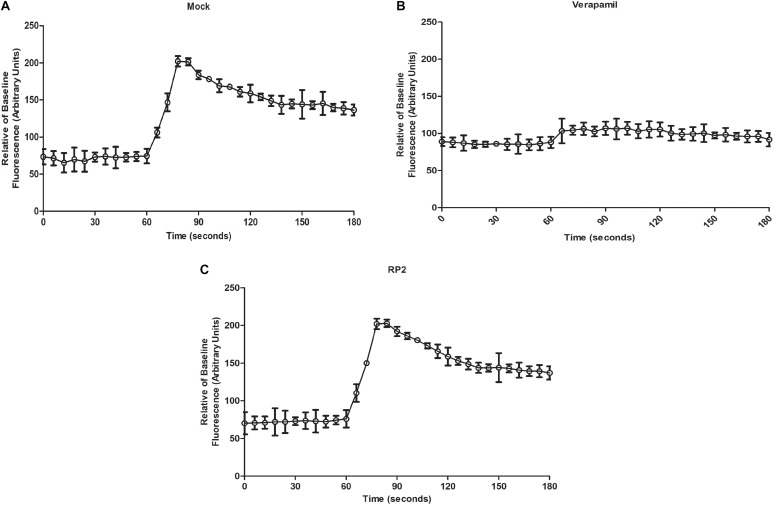
Mammalian calcium channel inhibition assays **(A)** Cytoplasmic Ca^2+^ measures of mock-treated cells with carbachol added at 60 s **(B,C)**. Same as panel **A** except that cells were treated with verapamil (50 μg/mL) **(C)** or RP2 (32 μg/mL), at 45 s pre-carbachol treatment. The results presented correspond to the mean of three independent readings ± SD.

We evaluated the effect of RP2 on human calcium channel activity by measuring the cytoplasmic Ca^2+^ levels using Fluo-4 dye. The endoplasmic calcium-channel activity was stimulated by the addition of carbachol which increased the Ca^2+^ levels by 2.6-folds into the cytoplasm (Figure 5A). On the contrary, the addition of verapamil abolished the Ca^2+^ ions accumulation inside the cytoplasm (Figure 5B). As shown in Figure 5C, HEK 293T treatment with RP2 at 32 μg/mL did not exhibit any significant effect on mammalian cell Ca^2+^ channel activity.

### *In vitro* Toxicity

**FIGURE 6:**
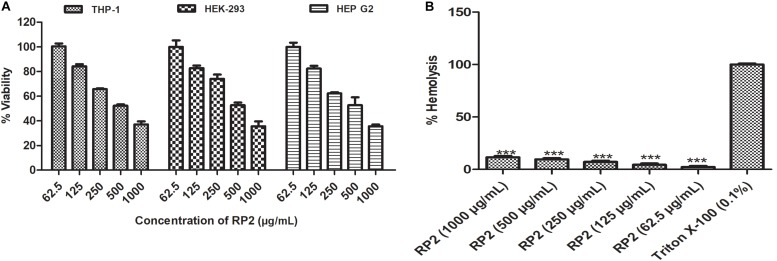
**(A)** Cytotoxic effect of RP2 on THP-1, HEK-293T, and HEP G2 cell lines. The cells were exposed to RP2 (1000–62.5 μg/mL) during 24 h at 37°C, 5% CO_2_. The results presented correspond to the mean of three independent readings ± SD. **(B)** Hemolytic effect of RP2 (1000–62.5 μg/mL) on rabbit erythrocytes. Triton X-100 (0.1%) was included as a positive control. The results presented correspond to the mean of three independent readings ± SD. Results were considered highly significant where ^∗∗∗^*P* < 0.001.

RP2 exhibited half-maximal inhibition concentration (IC_50_) at approximately 500 μg/mL for all the three cell lines tested (Figure 6A) and had negligible toxicity at the effective concentration (32 μg/mL). In hemolytic experiments, no significant lysis of red blood cells was observed up to 1000 μg/mL (Figure 6B).

### Macrophage Invasion Assay

**FIGURE 7:**
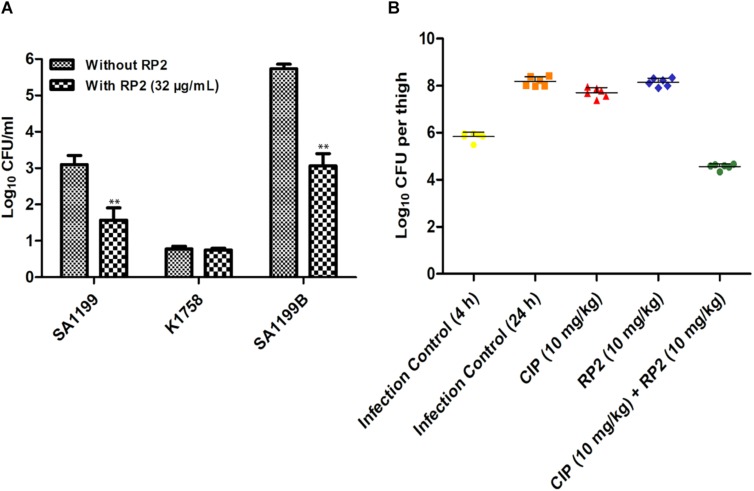
**(A)** Influence of RP2 (32 μg/mL) on the invasive abilities of *S. aureus* wild-type SA-1199, NorA over-producing *S. aureus* SA-1199B and the *norA* knock-out K1758. The results presented correspond to the mean of three independent readings ± SD. Results were considered significant when ^∗^*P* < 0.05 and highly significant when ^∗∗^*P* < 0.01 and ^∗∗∗^*P* < 0.001. **(B)** Neutropenic murine thigh infection model. Single dose (subcutaneous; 4 h after infection, 6 mice per group) treatment with ciprofloxacin and RP2 alone and in combination against *S. aureus* SA-1199B; for drug-treated animals, thigh CFU was determined at 20 h after infection. For controls, CFU in thighs was estimated at 4 and 20 h after infection. The CFU from each thigh are plotted as individual points and error bars represent the SD within an experimental group.

The intracellular invasion of *S. aureus* SA-1199B (NorA over-expressed) was nearly 2.5 log_10_ greater as compared to wild type SA-1199. *S. aureus* KA-1758 (*norA* gene deletion), attacked macrophages less effectively. In the presence of RP2 (32 μg/mL), the penetration of *S. aureus* KA-1758 remained unaffected, and whereas the invasiveness of *S. aureus* SA-1199B was reduced to a larger extent (1.5 log_10_) compared with the wild-type *S. aureus* SA-1199 (Figure 7A).

### *In vivo* Efficacy

We investigated the *in vivo* efficacy of ciprofloxacin in combination with RP2 using a neutropenic murine thigh infection model. We observed that neither ciprofloxacin (10 mg/kg) nor RP2 (10 mg/kg) alone indicated a significant reduction in bacterial counts (Figure 7B). However, the combination of ciprofloxacin and RP2 proved to be very efficient, resulting in a 1.3 and 3.5 log_10_ reduction in CFU, in comparison to the untreated control at 4 and 24 h, respectively (Figure 7B).

## Discussion

For bacterial infection, adherence, secretion of toxins and virulence factors, enzyme inactivation, efflux pumps, etc. are important mechanisms which cause bacterial pathogenicity and resistance to antibiotics. Thus to bowl over effects of pathogenicity in bacteria, identification of inhibitors to target these processes can be an effective strategy (Wang et al., 2014; Song et al., 2017; Imdad et al., 2018; Zhou et al., 2019). The efflux pumps contribute significantly to the development of multidrug resistance in clinical MRSA strains (DeMarco et al., 2007; Costa et al., 2013). The therapeutic failure of current antibiotics encourages the investigation of EPIs as adjunctive therapies. Several phytochemicals have been reported previously as EPIs (Khan et al., 2006; Kalia et al., 2012; Roy et al., 2013; Singh et al., 2017). The present study focused on the exploration of microbial diversity for the discovery of EPIs to combat multi-drug resistance. Our screening campaign identified *Streptomyces* Sp. IMTB 2501, whose crude fermentation extract displayed the characteristics of *S. aureus* efflux pump inhibition. Bioassay-directed fractionation yielded an indole metabolite, i.e., 2-(2-Aminophenyl) indole (RP2) previously unreported for the efflux pump inhibitory activity. Though indole derivatives have been reported for NorA efflux pump inhibition (Michalet et al., 2007; Ambrus et al., 2008; Buonerba et al., 2016), their toxicity or *in vivo* efficacy was not investigated.

RP2 demonstrated synergism with the tested antibiotics against efflux pump over-expressing *S. aureus* strains, reducing the MICs below their clinical breakpoints. We confirmed the EPI activity of RP2 in real-time fluorescence experiments using EtBr. Additionally, time-kill assay exhibited synergy because we observed >3 log_10_ reductions in CFU between the combination and most active counterpart (Belley et al., 2008). Moreover, RP2 reduced the frequency of resistant mutants in *S. aureus* and enhanced the PAE of all the tested antibiotics. Additionally, the *in silico* docking studies revealed that RP2 binds to active site of NorA efflux pump with a better docking score at the site 1. Comparative interaction analysis of RP2 and other NorA inhibitors like ferruginol, totarol, thioxanthene and phenothiazine docked to NorA suggests that they all target similar binding cleft and hence may adopt similar inhibitory mechanism (Bhaskar et al., 2016).

The ideal EPI should not dissipate the energy source of the pump by disrupting cytoplasmic membrane potential (Lomovskaya et al., 2001). Unlike CCCP, RP2 neither affected the membrane potential nor the total ATP production. Hence the synergistic activity observed was not a consequence of the off-target effect. Furthermore, RP2 had no significant cytotoxicity in mammalian cells, and unlike verapamil, it did not exhibit human Ca^2+^ channel inhibitory activities. These drawbacks have restricted the development of EPIs in the past (Blanchard et al., 2014). Inhibition of efflux pumps is also associated with other assaults in pathogenic bacteria such as reduced bacterial adherence (Kvist et al., 2008). As reportedly previously (Kalia et al., 2012; Singh et al., 2017), our results also corroborated the role of NorA over-production for the intracellular invasion of *S. aureus* in J774 macrophages. Involvement of RP2 as an EPI reduced the intracellular invasion. Similar observations have been reported earlier in MexAB-OprM over-producing *Pseudomonas aeruginosa* (Hirakata et al., 2009). So probably, *S. aureus* exports some factors required for the invasion using NorA efflux pump and RP2 reduces the invasion by inhibiting the NorA efflux system. The most prominent finding of this study was the efficacy of RP2 and ciprofloxacin combination in the murine thigh infection model.

## Conclusion

In conclusion, our docking studies combined with experimental evidences suggest that RP2 is a strong inhibitor of NorA that probably binds and blocks the active site/channel by sterically occluding export of antibiotics/drugs. RP2 represents a remarkable starting point for future medicinal chemistry-based development of lead candidates. The properties of RP2 may help deliver an adjunctive therapy to treat fatal bacterial infections.

## Data Availability

The datasets generated for this study, of the analyzed 16S rRNA gene sequence of strain IMTB 2501 can be found in GenBank under the accession number MK377074.

## Ethics Statement

This study was carried out in accordance with the recommendations of the Committee for the Purpose of Control and Supervision of Experiments on Animals (CPCSEA). The protocol was approved by the Institutional Animal Ethics Committee (IAEC/18/03) of the Institute of Microbial Technology, Chandigarh, India. Neither randomization nor blinding was considered necessary for the animal infection models.

## Author Contributions

RT and HN designed all the experiments, and wrote and edited the manuscript. RT, NM, NC, and MK performed all the microbiological experiments. RT, MJ, and NK performed the animal studies. SC and MR maintained the cell lines and contributed to the cytotoxicity experiments. SJ contributed to the NMR spectroscopy and structure elucidation. DV and KT performed the *in silico* docking studies. RT, MJ, and HN analyzed the results. RT and HN wrote and edited the manuscript. All the authors approved the manuscript before submission.

## Conflict of Interest Statement

The authors declare that the research was conducted in the absence of any commercial or financial relationships that could be construed as a potential conflict of interest.
