# Supplementary material for: Microbe-Derived Indole Metabolite Demonstrates Potent Multidrug Efflux Pump Inhibition in Staphylococcus aureus
Source: Front Microbiol. 2019 Sep 18;10:2153. doi: 10.3389/fmicb.2019.02153 (PMC6759831; doi:10.3389/fmicb.2019.02153)
Supplement: Supplementary file 1 [file Data_Sheet_1.PDF]

Supplementary Material

Supplementary Methods

S.1 Bacterial culture and extract library preparation

A library of 4000 microbial isolates was explored for the EPI activity. The crude fermentation extract was prepared in 50 mL of CSPY (casein enzyme hydrolysate 10%, soluble starch 10%, peptone 1% and yeast extract 1%) medium at 30°C, 200 rpm for 3-4 days. The cell-free supernatant was mixed with pre-activated Diaion® HP-20. After 3-4 hours of intermittent mixing, the resin was filtered from the supernatant and then desalted by rinsing with Milli-Q water. Metabolites were eluted from the resin in 100 mL of methanol: isopropanol: acetone (7:2:1) mixture. The organic solvent was evaporated *in vacuo*. Dried extracts were resuspended in 2 mL of dimethyl sulfoxide (DMSO) and stored at -80°C, until bioactivity assessment.

S.2 Screening of microbial extract library for EPI activity and identification of producer strain

The MICs (minimum inhibitory concentrations) of antibiotics, as well as extracts, were determined using standard microdilution assay (Wayne, 2011). The EtBr modulatory activity of extracts was evaluated using 3-(4, 5-dimethylthiazol-2-yl)-2, 5-diphenyltetrazolium bromide (MTT) assay. Test strains (NorA, TetK, and MsrA) were incubated in the presence of extracts (at sub-inhibitory concentrations; 1/4th of MIC) and EtBr (in a 2-fold serial dilution manner). Extracts that enhanced the effect of the EtBr in one or more test strains of MDR *S. aureus* were further evaluated in the presence of various antibiotics to confirm the EPI activity. Further, genomic DNA of the most potent strain (IMTB 2501) was extracted using ZR Fungal/Bacterial DNA MiniPrep™ (Zymo Research) kit. Genetic Analyzer ABI3130XL (Applied Biosystems, USA) was used for 16S rRNA gene sequencing.

Supplementary Tables

TABLE S1. Extracts having modulation factor (MF) ≥ 2 with ethidium bromide (EtBr) against various efflux pumps over-expressed *Staphylococcus aureus* strains

IMTF2878	IMTB2478	IMTB2270	IMTF2146	IMTF947
MTCC7154	IMTB2479	IMTB2275	IMTF3086	IMTF935
MTCC9313	IMTF2454	IMTB2273	IMTF2143	IMTF1206
MTCC6546	IMTF2452	IMTB2336	IMTB3037	IMTF2878
MTCC10622	IMTB2501	IMTF999	IMTF1862	IMTF971
IMTF2527	IMTB2335	IMTF998	IMTB2042	IMTB1671
IMTF2525	IMTB2342	IMTB2214	IMTB2084	IMTB1224
IMTB2581	IMTB2414	IMTB2261	IMTF1118	FIMT1010
IMTF2430	IMTB2421	IMTF981	IMTF1984	DSF44
IMTF2413	IMTF2338	IMTF997	IMTF1950	IMTY809

TABLE S2. Modulation effect of shortlisted extracts with Norfloxacin, Tetracycline and Erythromycin against various efflux pumps over-expressed *Staphylococcus aureus* strains

**Sr. No.**	**Extract Name**	**MIC of crude extract (µg/ml)**	**Concentration of an extract used for modulation (µg/ml)**	**Modulation Factor (MF)**		
				**SA-1199B[1]**	**XU212[2]**	**RN4220[3]**
				**Norfloxacin**	**Tetracycline**	**Erythromycin**
1.	IMTB2501	500	125	16	16	16
2.	IMTF2454	500	125	8	4	-
3.	IMTF2413	250	64	4	2	-
4.	IMTB2342	250	64	4	2	2
5.	IMTF2261	250	64	4	-	-
6.	IMTF1118	250	64	16	8	4
7.	IMTF1984	250	64	4	2	-
8.	IMTF935	500	64	4	-	2

[1]*S. aureus* with NorA Pump over-expression; [2]*S. aureus* with TetK Pump over-expression; [3]*S. aureus* with MsrA Pump over-expression

TABLE S3. ^{1}H (400 MHz) and ^{13}C (100 MHz) NMR Data (δ in ppm) of the compound RP2

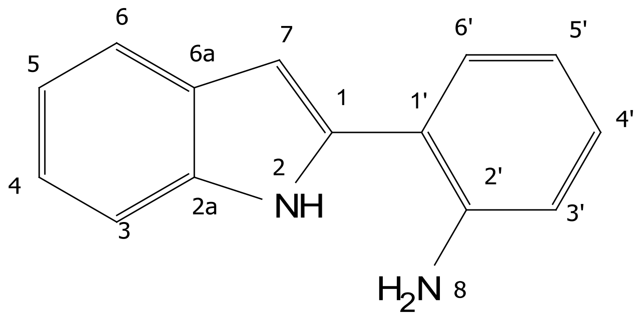

No.	δ_C, type	δ_H, mult.[a] (*J* in Hz)
1	136.71 (C)	
1´	117.51(C)	
2		11.24 (s, 1H, N*H*)
2a	136.55 (C)	
2´	146.07 (C)	
3	111.58 (*C*H)	7.4 (d, 1H, *J*=7.96)
3´	117.03(*C*H)	6.70 (dd, 1H)
4	128.83(*C*H)	7.06 (ddd, 1H)
4´	116.21(*C*H)	6.83 (dd, 1H, *J*=8.04)
5	119.51(*C*H)	6.99 (ddd, 1H, *J*=7.44 Hz)
5´	121.49(*C*H)	7.09 (ddd, 1H)
6	120.19(*C*H)	7.53 (d, 1H, *J*=7.72 Hz)
6a	129.08 (C)	
6´	129.24(*C*H)	7.36 (dd, 1H)
7	100.41(*C*H)	6.68 (ddd, 1H)
8		5.19 (s, 2H, NH_2)

[a]some peaks are overlapped, and therefore multiplets may vary.

TABLE S4. Results of modulation assays for reserpine

**Test Compound (µg/ml)**	**MIC (µg/ml) of antibiotics for indicated strain with/without reserpine (fold modulation)**		
	Norfloxacin SA-1199B (NorA)	Tetracycline XU212 (TetK)	Erythromycin RN4220 (MsrA)
Reserpine (20)	32/4 (8)	128/32 (4)	128/128 (-)

Supplementary Figures

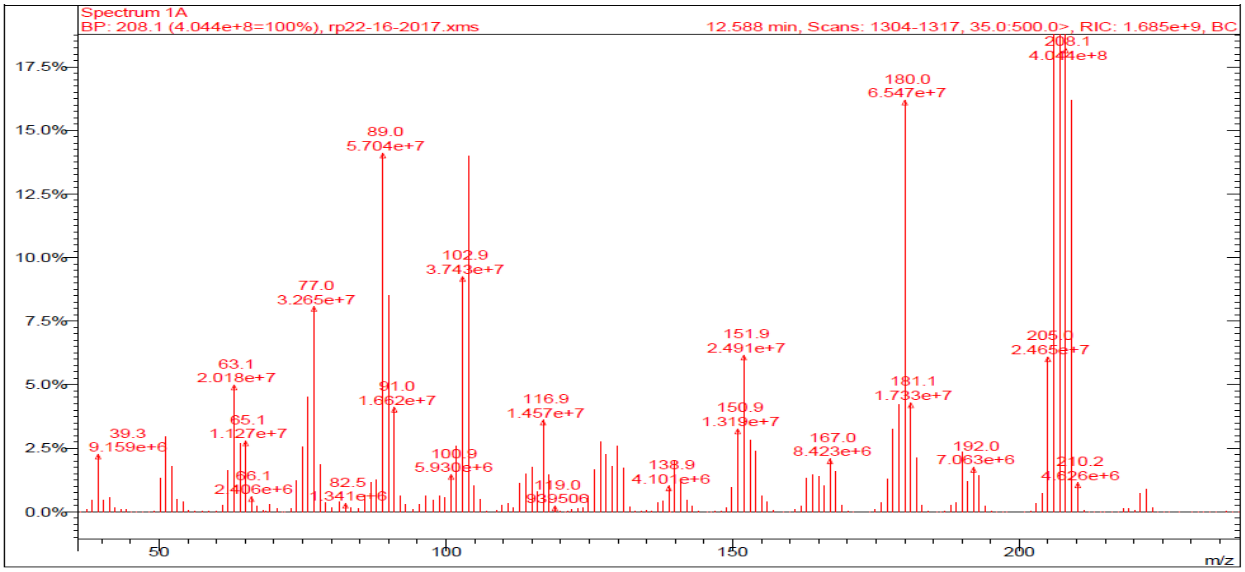

FIG. S1 GC-MS spectrum of RP2

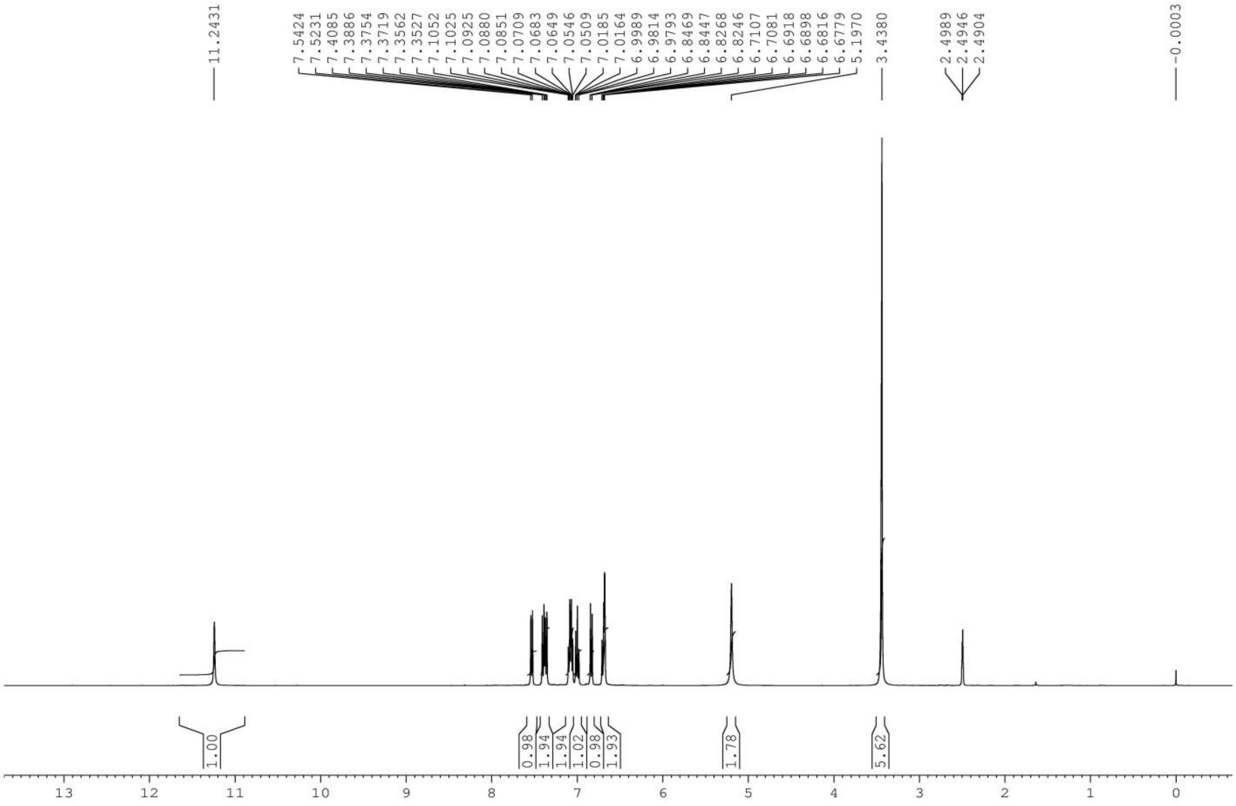

FIG. S2 ^{1}H NMR spectra of RP2

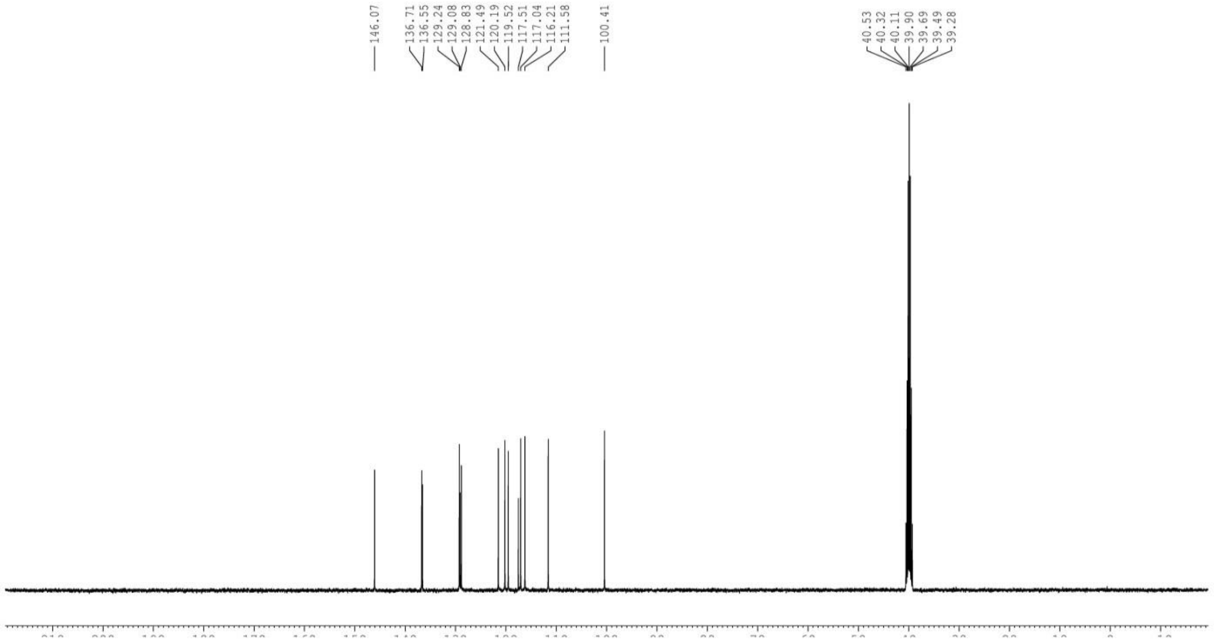

FIG. S3 ^{13}C NMR spectrum of RP2

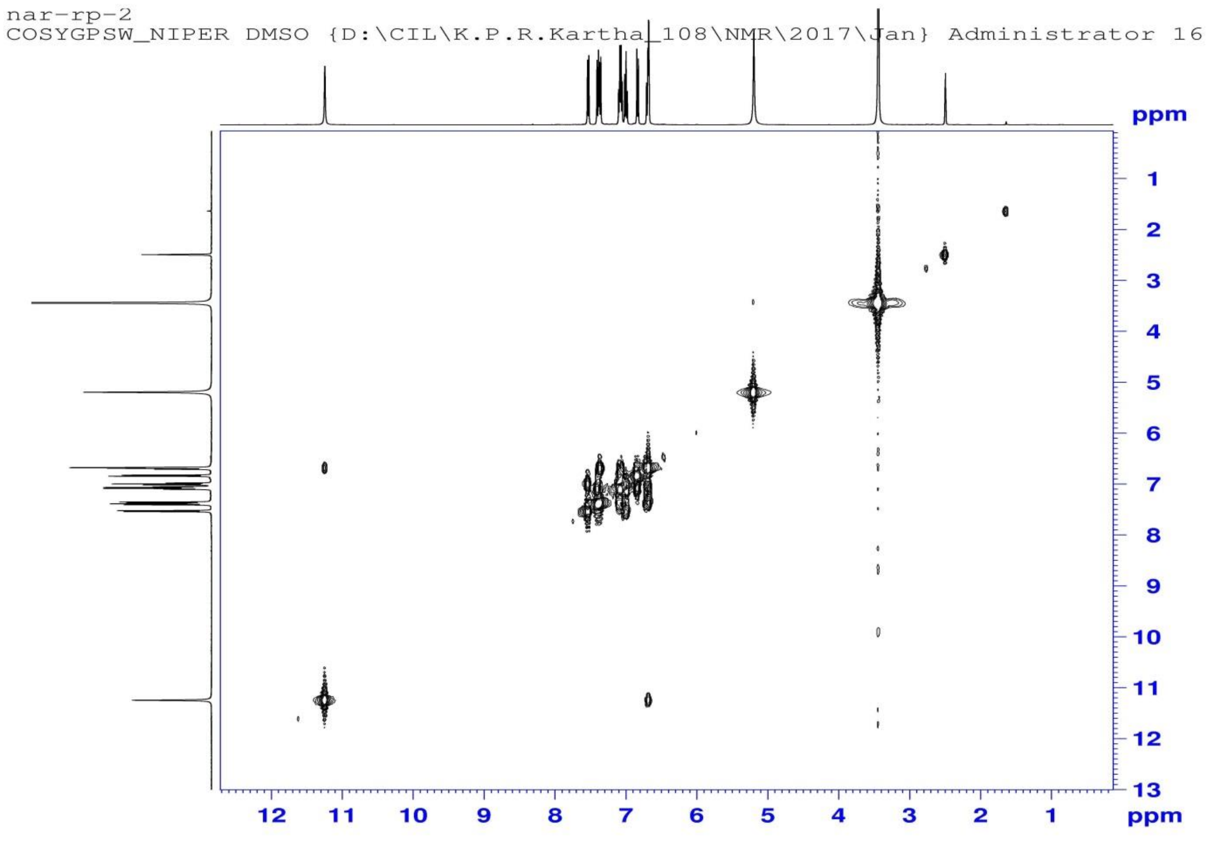

FIG. S4 2D COSY NMR spectrum of RP2

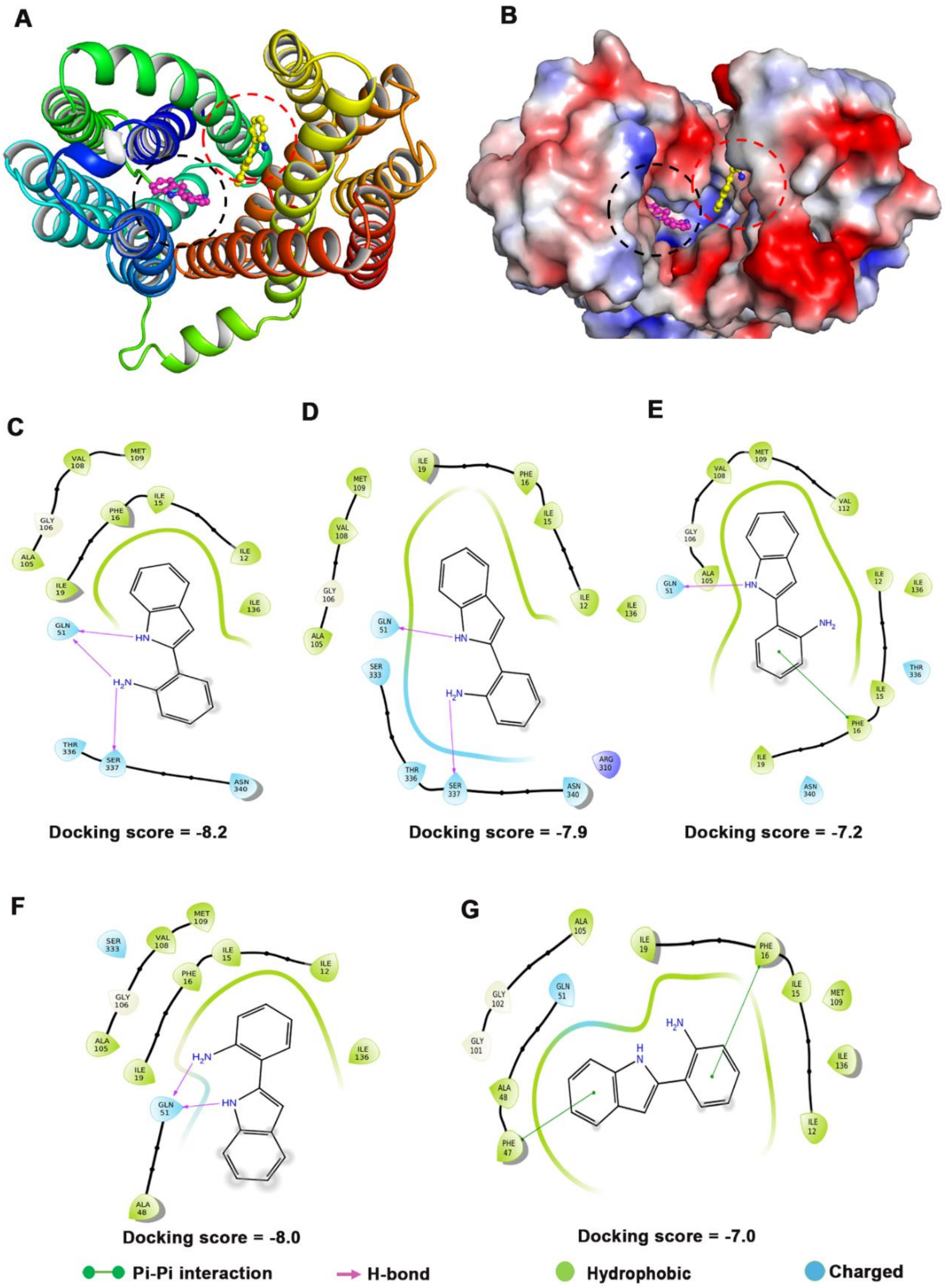

FIG. S5 *In silico* docking studies. (A and B) Two distinct sites, site 1 and site 2, where RP2 docked in the NorA active site are shown in black and red broken circles respectively. Docked RP2 molecules are shown in ball and stick representation in magenta and yellow colors at site 1 and site 2, respectively. 57 poses were observed at the site 1 while only 8 poses, having comparatively lower docking scores, were observed at site 2. (C to G) Ligand interaction diagrams in 2D representation

for the five best docking poses of RP2 at site 1 of predicted NorA structure. The interacting residues and binding clefts were mostly conserved however, as expected there were some minor differences in the nature of binding interactions in different poses.

References

Wayne, P. (2011). Clinical and laboratory standards institute. Performance standards for antimicrobial susceptibility testing.
